# Experimental Study on Metal Parts under Variable 3D Printing and Sintering Orientations Using Bronze/PLA Hybrid Filament Coupled with Fused Filament Fabrication

**DOI:** 10.3390/ma15155333

**Published:** 2022-08-03

**Authors:** Xueying Wei, Ingolf Behm, Tony Winkler, Stefan Scharf, Xujun Li, Rüdiger Bähr

**Affiliations:** 1 Institute of Manufacturing Technology and Quality Management, Otto-von-Guericke-University Magdeburg, Universitätsplatz 2, 39106 Magdeburg, Germany; ingolf.behm@ovgu.de (I.B.); tony.winkler@ovgu.de (T.W.); stefan.scharf@ovgu.de (S.S.); xujun.li@ovgu.de (X.L.); ruediger.baehr@ovgu.de (R.B.); 2 State Key Laboratory of Multiphase Flow in Power Engineering, Xi’an Jiaotong University, Xianning West Road 28, Xi’an 710049, China

**Keywords:** FFF additive manufacturing, printing and sintering directions, bronze/PLA hybrid filament, shrinkage, mechanical property, porosity

## Abstract

Producing metal parts from Fused Filament Fabrication (FFF) 3D printing coupled with a metal/polymer hybrid filament, considering the advantages of high-performance and low cost, has generated considerable research interest recently. This paper addresses the studied relationship between variable printing/sintering directions and the properties of the sintered metal parts. It was shown that the printing directions played a significant role in determining the properties of final products, such as shrinkage, tensile stress, and porosity. The shrinkage in the layer direction because of anisotropic behavior is more minor than in the other dimensions. The microstructural analysis indicated that the printing directions had influenced the form and position of porosity on the produced metal parts. Most porosities occurred on the surfaces printed parallel to the printing bed. Furthermore, the sintering orientations had no possible benefits for dimension shrinkage, weight shrinkage, density, and porosity position of produced metal parts. However, the sintering direction “upright” resulted in parting lines inside the sintered tensile samples and made them fragile. The best printing-sintering combination was “on-edge-flat”.

## 1. Introduction

Metal injection molding (MIM), due to good precision and adequate surface quality, is a promising method for manufacturing metal parts. It is widely used in many industries, such as mechanical engineering, the automotive industry, aerospace, the electrical industry, etc. [1,2,3,4,5,6,7,8]. Nevertheless, the process has some disadvantages that stand in the way of economical and agile production. Moreover, it is sometimes difficult to produce metal parts with complex structures. At this point, additive manufacturing can compensate for the limitations of the MIM process; for example, using 3D printing with fused filament fabrication (FFF) and metal/polymer hybrid filament to produce more complex metal parts. In addition to high degrees of geometric freedom and maximum flexibility, additive manufacturing using metal/polymer hybrid filament enables very cost-effective and rapid manufacturing [9,10,11] and has generated considerable recent research interest [10,12,13,14,15,16]. However, the properties of sintered metal parts were far from optimal, especially shrinkage, density, tensile stress, and porosity. Therefore, the analysis and optimization of FFF 3D printing-produced metal parts are desired.

Over the years, numerous experiments have been conducted to study the manufacturing process of metal parts using metal/polymer composite filament. Both Godec et al. [17] and Fafenrot et al. [18] reported that the modification of printing parameters, such as decreasing layer thickness, increasing nozzle diameter, controlling infill percentage, infill orientation and infill pattern, etc., had a significant influence on the final tensile properties and the porosity formation. Moreover, by expanding the infill percentage and reducing layer thickness, the tensile stress could be improved by 17%. Gong et al. [13], Caminero et al. [19] and Kurose et al. [20] observed that the more considerable shrinkage often occurs in the layer direction because of the influence of gravity. Caminero et al. [19] and Kurose et al. [20] investigated the effect of printing directions on the final properties of the metal parts using three printing orientations: “flat”, “on-edge”, and “upright”. The results showed that the “on-edge” printing direction has the optimum mechanical performance. Burkhardt et al. [12] and Thompson et al. [21] noticed large volume porosity of the metal parts and that the porosity could be reduced by extending the sintering holding time. On the other hand, Caminero et al. [19] and Liu et al. [16] also reported a low porosity content of <8%.

Generally, there are three types of debinding processes: catalytic debinding [8,19], solvent debinding [8,11,22], and thermal debinding [3,5,8,14,23]. In the catalytic debinding process, the binder polyacetal (POM) is broken down into formaldehyde using gaseous nitric acid. During the process, the binder becomes gaseous and leaves the green part without a liquid phase. However, this is a high-cost process which requires a special debinding machine. Through the solvent debinding process, the soluble binder is extracted in a chemical solvent, such as ethanol, acetone, etc. The backbone, which supports the part’s geometry, is vaporized at the beginning of sintering. In the thermal debinding process the green parts are embedded in the sand to support the geometry during the debinding and sintering processes. This is different from catalytic and solvent debinding as it does not require any additional support. The gaseous nitric acid and chemical solvent were not noticed to be factors in the mentioned works. The only significant factors were temperature and sand, which made the thermal process the greenest and cheapest method for FFF metal parts production. Given that the support provided by sand was a significant factor, it would be expected that sintering directions in conjunction with printing directions would also be influential.

Unfortunately, few papers have addressed the thermal debinding/sintering process. Moreover, these previous works have not focused on analyzing the influence of the printing directions in combination with different sintering directions [12,15,16,19,21] on the properties of metal parts. These methods have only discussed the sintered parts from different printing orientations rather than the metal parts from various sintering directions. Further work is required to investigate the mechanical and physical properties as well as porosity caused by the different printing/sintering processes.

This study focuses on the thermal debinding/sintering process. The purpose being to describe and examine the separate printing orientations and united sintering orientations by using bronze/PLA hybrid filament coupled with the FFF 3D printing process. In this study, we observed the printing/sintering orientations effects on the metal parts physical/mechanical properties and porosity. In this paper we determined the influence of the sintering directions on the shrinkage and density by analyzing cubes. Furthermore, we define the effect of variable printing/sintering directions on tensile stress and porosity by using tensile specimens. The study introduced unusual shrinkage in layer dimensions, invariable density under varying orientations, and researched the best printing/sintering combination of final metal parts. The position and volume of porosity, both on the surface and inside of metal parts, from divergent printing/sintering orientations are illustrated.

## 2. Materials and Methods

### 2.1. Materials

The material used in this study was the bronze/PLA hybrid filament from *The Virtual Foundry Company* (85 wt. % bronze powder, 15 wt. % PLA, and trace additive binder). The components of bronze/PLA hybrid filament are shown in Table 1.

### 2.2. 3D Printing

The green part was printed by using a Prusa i3 MK3 desktop 3D printer. The printing process of the green part was the same as regular PLA FFF printing. The printing parameters were based on standard printing settings from PrusaSlicer version 2.3.0 (Prusa Research, Czechia Republic). In consideration of the metal powder flowing through the nozzle during the printing process, a steel nozzle was used during the experimental trials as it is a harder material. The nozzle diameter was used to match the nozzle’s larger diameter (0.6 mm). Due to the high nozzle diameter, the layer thickness was increased to 0.3 mm to avoid any possible blockage during the printing process. The printing parameters are as shown in Table 2.

### 2.3. Thermal Debinding and Sintering

**Figure 1:**
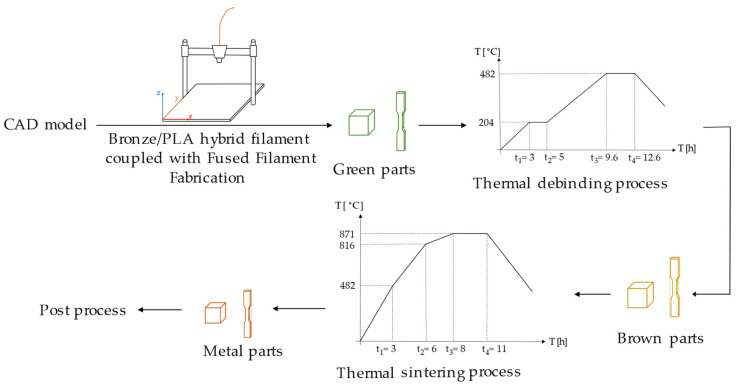
Scheme of bronze/PLA hybrid filament coupled with FFF process.

The debinding and sintering were performed using a thermal process. The process started with the debinding process by heating the green parts to 204 °C for 2 h and then slowly heating them to 482 °C in 3 h. Afterward, the sintering process was carried out by slowly heating the brown parts at a temperature of 871 °C for 3 h. It should be noted that the debinding and sintering processes took place in an open environment. Moreover, the green parts were embedded in sand inside an alumina crucible to avoid the geometry change during the gasification process due to the high temperature. Furthermore, the green parts were covered by superfluous carbon powder to prevent any possible oxidation of the final produced metal parts. A schematic drawing of the production process of the bronze/PLA hybrid filament coupled with FFF process can be seen in Figure 1.

### 2.4. Printing and Sintering Direction

#### 2.4.1. Printing and Sintering Direction for Cubes

**Figure 2:**
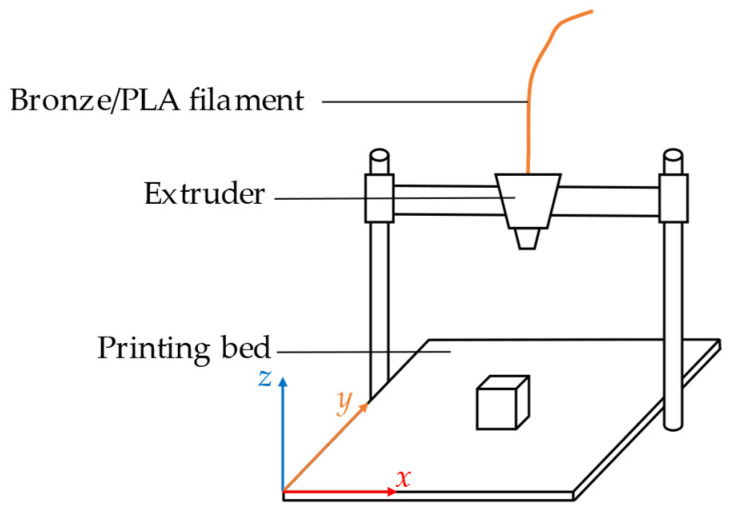
Printing direction of cubes with edge length 10 mm.

To analyze the effects of sintering directions on the dimension shrinkage, weight shrinkage, and density we printed cubes with an edge length of 10 mm for the first experiment. A cube has the same dimensions on the *x*-, *y*-, and *z*-axes. Using this shape simplifies the task of observing and comparing any changes in the size and weight of the three dimensions before and after sintering. The cubes in this experiment were printed using a cartesian coordinate system, as shown in Figure 2.

**Figure 3:**
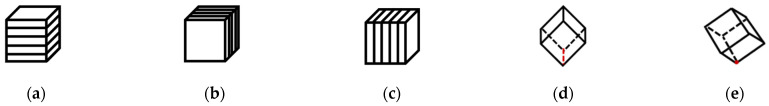
Sintering directions of cubes and their illustrations: (**a**) on *xy* surface; (**b**) on *xz* surface; (**c**) on *yz* surface; (**d**) on an edge; (**e**) on a point.

In the sintering process there are many sintering directions to choose from that influence how the part is supported in the sand. Five sintering directions are shown in Figure 3. The blocks stand on the *xy*, *xz*, and *yz* surfaces; on an edge; and on a point of the cube, respectively.

#### 2.4.2. Printing and Sintering Directions for Tensile Specimens

**Figure 4:**
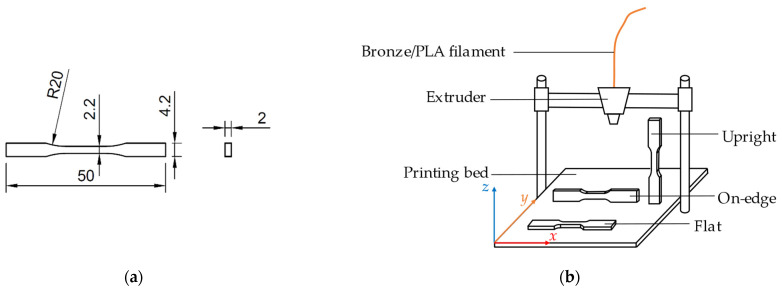
Schematic drawing of the printing process of the investigated specimens: (**a**) actual dimensions of the tensile test sample in (mm); (**b**) printing directions of the tensile simples.

The second experiment was to study the effect of printing and sintering directions on tensile specimens. The standard of tensile specimens used in the investigation comes from DIN EN ISO 527-2: 2012-06,1BB [24], displayed in Figure 4a. The tensile specimens were printed in three directions (flat, on-edge, and upright) as depicted in Figure 4b.

Nine different combinations were obtained by combining three printing directions and three sintering directions (see Table 3 for details).

### 2.5. Shrinkage and Density Analysis

The *x*, *y*, and *z* dimensions of the cube specimen were measured with a caliper gauge before and after sintering. The shrinkage of each dimension was then calculated. The density of the cube was measured using a densimeter Mk2200 (MK Industrievertretungen GmbH, Stahlhofen am Wiesensee, Germany). The density (ρ) of the sample was confirmed by the mass of the sample in the air ($m_{s}$), the density of the embedded liquid ($\rho_{l}$), gravitational acceleration (g), and the mass of the specimen in the liquid ($m_{l}$). The calculation of density is shown in the following Equation (1):

(1) $$\rho =m_{s}\times \rho_{l}\times g/m_{l}$$

### 2.6. Microstructural Characterization

The microstructural investigation of the produced parts was performed using an optical microscope and a scanning electron microscope (SEM). The images of the surface structure and cross-section of the green part were taken by a FEI XL30 ESEM (Thermo Fisher Scientific Inc., Waltham, MA, USA). The cross-sections of metal parts (length 20 mm, width 3 mm, thickness 1.5 mm) were ground, polished, and investigated using a KEYENCE VHX-5000 digital microscope (Keyence Corporation of America, Elmwood Park, NJ, USA). In addition, the volume fraction of the porosity was measured using ZEISS image analyzer software (Carl ZEISS Microscopy GmbH, Jena, Germany).

### 2.7. Mechanical Characterization

The tensile test specimens (see Figure 4a) were evaluated by a TT28100 universal testing machine (TIRAtest GmbH, Schalkau, Germany). The flat specimens had a thickness of 2.0 mm and a gauge length of 10.2 mm. The tensile test followed DIN EN ISO 6892-1: 2020-06 [25], and the traverse speed of the tensile machine was set to 1 mm/min.

## 3. Results and Discussion

To obtain the most reliable experimental results each experiment group contained five parallel specimens. The results were discussed in the following points.

### 3.1. Shrinkage and Density Analysis through Sintered Cubes

**Figure 5:**
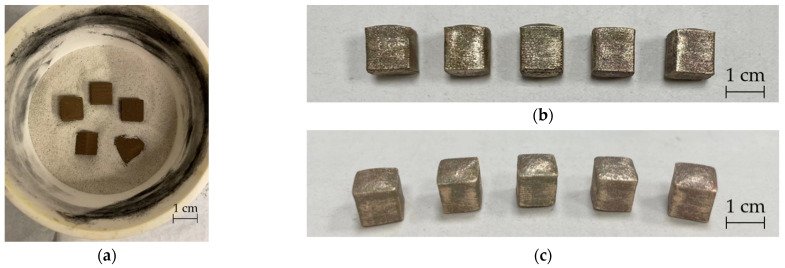
Sintered cubes according to five sintering directions before and after sintering: (**a**) cubes according to five sintering directions were embedded in sand; (**b**,**c**): cubes after sintering (from left to right: sintering on *xy* surface, on *xz* surface, on *yz* surface, on an edge, and on a point).

Figure 5a shows that the cubes were embedded and sintered in sand. Five cubes before (Figure 5a) and after sintering (Figure 5b,c).

The volume of the green part cube was 1 cm^3^ (1000 mm^3^), and the weight was generally 4 g (±5%). In Table 4 the weight, shrinkage, and density increase were obtained for the dimensional shrinkage of the *x*-, *y*-, and *z*-axes. The shrinkage of the *x*- and *y*-axes was between 20% and 21% and the shrinkage of the *z*-axis was between 11% and 14%. After sintering the mass loss of the specimen was about 15%, which also confirmed the mass fraction of bronze is 85%. Additionally, the density increased from 3.7 g/cm^3^ to about 6.6 g/cm^3^. The results showed that the issues from the five sintering orientations were generally similar and there was no significant influence from the five sintering directions on the shrinkage and density of sintered parts.

**Figure 6:**
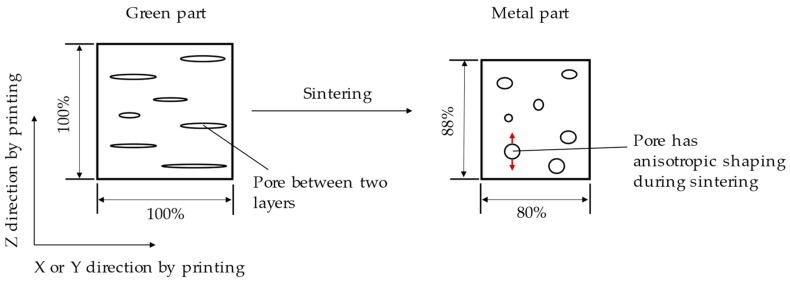
Anisotropic behavior for shrinkage caused by the change of pore structure during the sintering process.

According to Table 4 the shrinkage of the *z*-axis was smaller than that of the *x*- and *y*-axes because there were pores between the layers in the green part. This same phenomenon was deeply analyzed by the authors of [26] and a reasonable explanation was proposed. After printing the pores were formed flat between layers (Figure 6). During the process of sintering under high temperatures the pores showed anisotropic behavior changing from a plane shape to a spherical shape (Figure 6). The uniaxial forming of the pores was opposite to the sintering shrinkage direction on the *z*-axis, so the shrinkage was less than that on the *x*-axis and the *y*-axis. Consequently, the porosity generated during printing affected the dimension shrinkage.

The density of specimens increased significantly after sintering, as Table 4 showed. While the standard density of bronze is 8.8 g/cm^3^ [27,28,29], the density of the metal parts was still lower due to the existence of the pores. After sawing the specimens large pores were observed. The presence of pores leads to a significant reduction in the density of metal parts.

### 3.2. Tensile Stress and Porosity Analysis through Tensile Specimens

**Figure 7:**
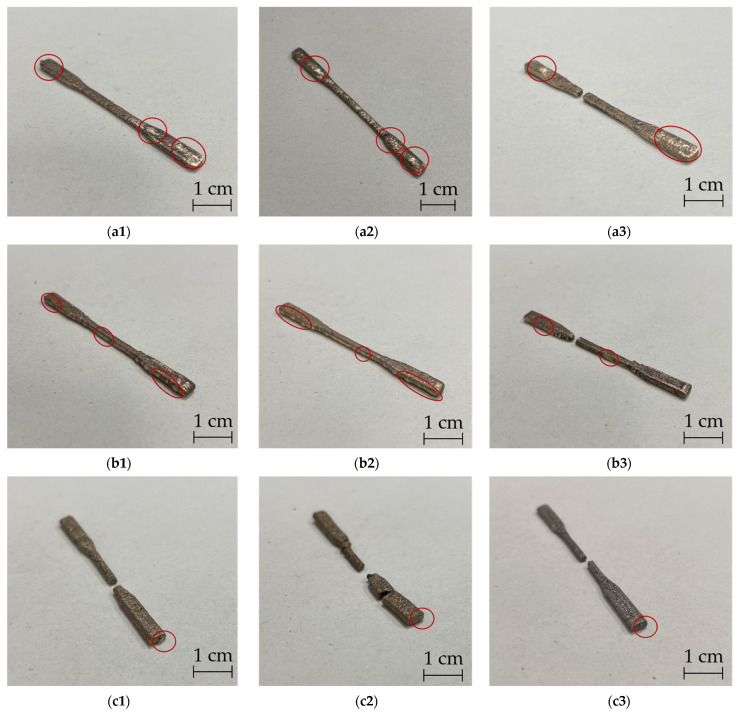
Results of pore position on metal tensile specimens from nine combinations between printing directions and sintering directions (the red circles represented the pores on surfaces of metal parts): (**a1**) flat printing-flat sintering; (**a2**) flat printing-on-edge sintering; (**a3**) flat printing-upright sintering; (**b1**) on-edge printing-flat sintering; (**b2**) on-edge printing-on-edge sintering; (**b3**) on-edge printing-upright sintering; (**c1**) upright printing-flat sintering; (**c2**) upright printing-on-edge sintering; (**c3**) upright printing-upright sintering.

Figure 7 shows sintered tensile specimens in nine combinations between printing and sintering directions.

#### 3.2.1. Tensile Stress Analysis

**Figure 8:**
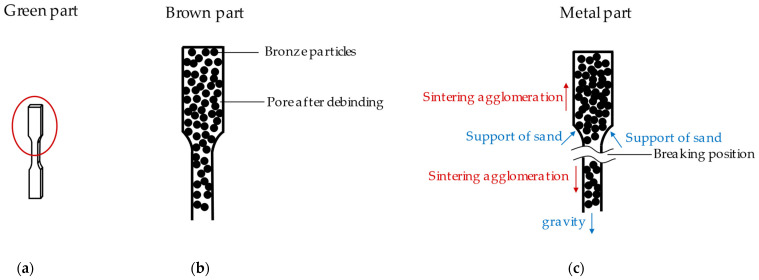
Diagram of fracture mechanism of vertical sintered parts: (**a**) green part; (**b**) brown part. PLA vaporized and only bronze particles remained in the part; (**c**) metal part. Material fracture caused by uneven distribution of sintering concentration, gravity, and sand support effect.

It was observed that all specimens with printing and sintering direction “upright” were broken after the sintering process. It is well-known that the interconnection between layers of green parts from additive manufacturing is weak [30,31,32]. For the specimens printed in the “upright” orientation the number of layers was higher than in the other two printing directions. Any sample that had fewer combinations between layers were broken after sintering. Some parts broke before the end of the entire printing process. During the debinding and sintering processes the specimens were embedded in the sand, which supported the specimens. In the sintering process metal particles flowed and produced sintering agglomeration, in accordance with the findings of the authors of [33,34]. In this case, the standing specimen (Figure 8a) formed its agglomerations at the top and in the middle, discharging the particles up or downward (changing shown from Figure 8b to Figure 8c). In addition, the gravity of the specimens and the support from the sand also played essential roles. Due to the reduction in material and separation force at this position the specimens broke at the location marked in Figure 8c.

**Figure 9:**
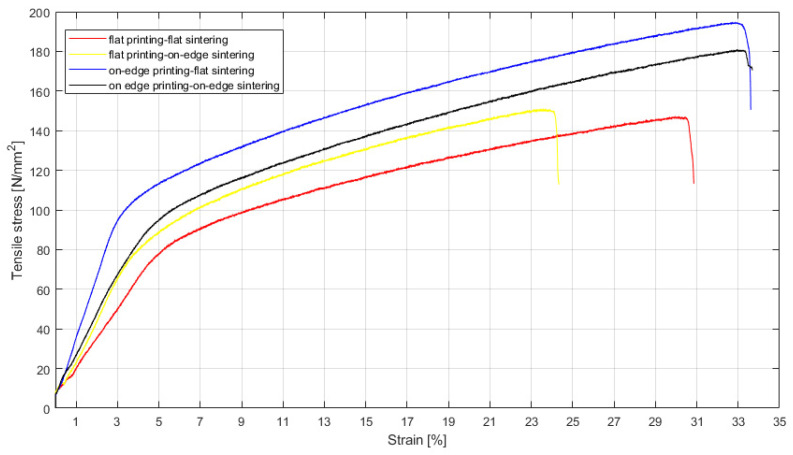
Tensile stress–strain curves of metal parts from test No. 1, 2, 4, and 5 in Table 3.

After sintering only the specimens from four printing-sintering combinations (flat printing-flat sintering, flat printing-on-edge sintering, on-edge printing-flat sintering and on-edge printing-on-edge sintering) were successfully sintered (Test No. 1, 2, 4 and 5 in Table 3). These parts were tested for their mechanical properties. Figure 9 below shows the determined stress–strain curves. As can be seen from the graph only slight differences in tensile stress were determined between the same printing directions. However, the tensile stress of printing direction on-edge was higher than in the flat-printing direction. Since the porosity influences, the tensile stress, and the pore volume on the surface of the on-edge-printed specimens were smaller than those of plane printed samples (see Figure 7a,b) the on-edge-printed specimens had better tensile stress. Thus, porosity played a key role in tensile stress. Considering the determined porosity volumes on the surface and the tensile stresses of all variants produced the combination of printing and sintering “on-edge-flat” can be identified as the best.

#### 3.2.2. Microstructural and Porosity Analysis

**Figure 10:**
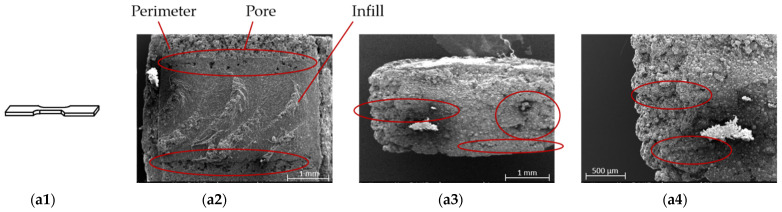

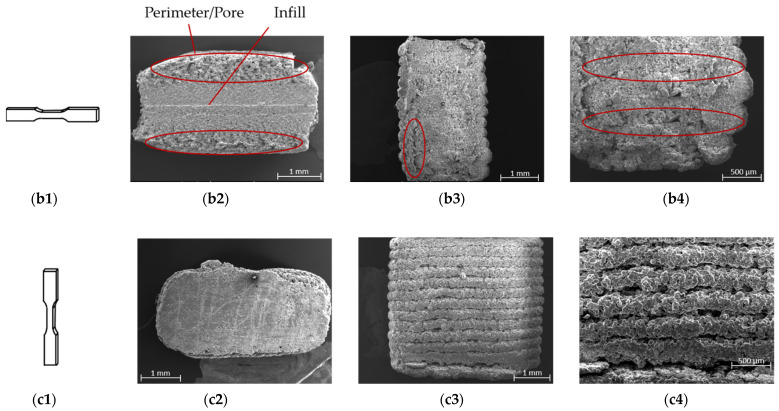
Illustration and scanning electron microscope (SEM) images of printed green tensile specimens (The red circles showed pores/gaps on the surfaces or inside of the specimens). (**a1**) Illustration of flat printed specimen; (**a2**) top view of the flat specimen; (**a3**) cross section of the flat specimen; (**a4**) zoomed view of cross section of the flat specimen; (**b1**) illustration of on-edge printed specimen; (**b2**) top view of the on-edge specimen; (**b3**) cross section of the on-edge specimen; (**b4**) zoomed view of cross section of the on-edge specimen; (**c1**) illustration of upright printed specimen; (**c2**) top view of the upright specimen; (**c3**) cross section of the upright specimen; (**c4**) zoomed view of cross section of the upright specimen.

According to Figure 7 most of the pores were located on the layers parallel to the printing bed during the printing process. However, there were only a few or no pores on the layers that were positioned perpendicular to the print bed. The green tensile specimens with different printing directions were subject to supplementary SEM examinations. Figure 10 shows the illustrations of three printing directions (Figure 10(a1–c1)), top views (Figure 10(a2–c2)), and cross sections (Figure 10(a3–c4)) of the green parts. The flat printed and on-edge printed samples had fewer layers than the “upright” printing direction. According to Figure 10(a2–c2) the infill in the layers was bonded tightly. There was no gap between the infills. Only a few small pores were detected in the sample cross-section, as Figure 10(a2–c2) shows. On the other hand, there were big gaps between layers from the cross section of green parts shown in Figure 10(a3–c4).

**Figure 11:**
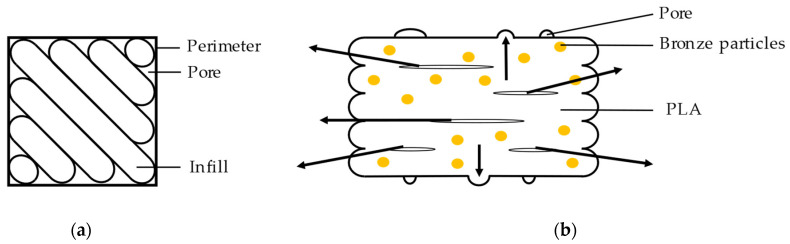
Diagram of PLA volatilization and pore formation during debinding: (**a**) top view of a green part; (**b**) cross section of a green part and the exit directions of gaseous PLA during debinding.

As the evaluation of Figure 10 showed the interconnection between infill is much larger than an interconnection between layers inside of green specimens (indicated in Figure 11) [20]. During the debinding process the gaseous PLA could diffuse out of the part through the gaps between layers. The tight interconnection of the filler in the layers prevents the gaseous PLA from diffusing out of the metal parts to a certain extent causing pores to be formed. The experimental results (see Figure 7) showed that the pore volume of flat printed specimens was the largest and that of the upright printed specimen was the smallest, which is a function of the area of the specimen parallel to the printing bed.

**Figure 12:**
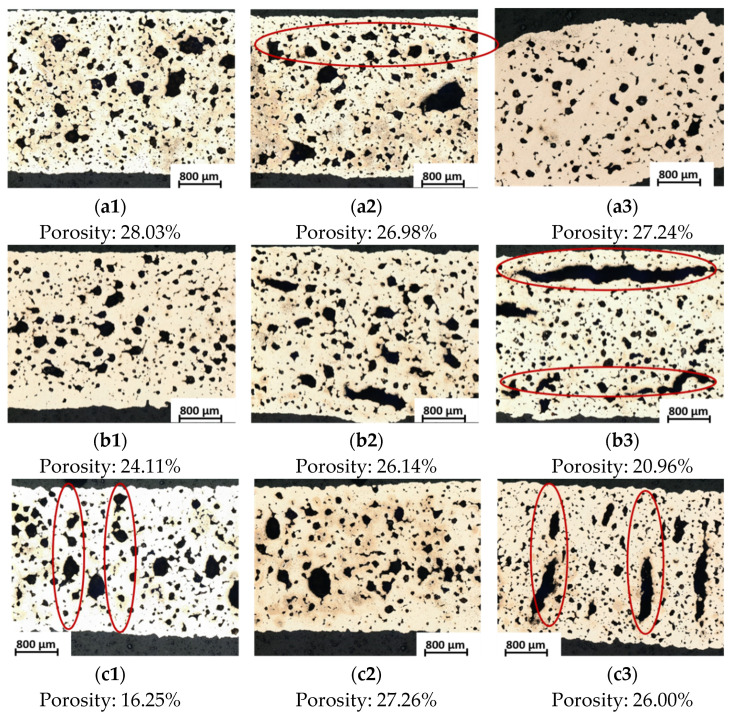
Microstructure and porosity volume fraction of different combination specimens in printing and sintering directions (the red circles represented the shapes built from pores): (**a1**) flat printing–flat sintering; (**a2**) flat printing–on-edge sintering; (**a3**) flat printing–upright sintering; (**b1**) on-edge printing–flat sintering; (**b2**) on-edge printing–on-edge sintering; (**b3**) on-edge printing–upright sintering; (**c1**) upright printing–flat sintering; (**c2**) upright printing–on-edge sintering; (**c3**) upright printing–upright sintering.

The microstructure and porosity volume fraction of the nine metal specimens appears in Figure 12. Inside of metal parts, the pores were imaged clearly. It is worth mentioning that, while the form of pores was diverse, the volume fraction of porosity inside of the parts was similar in each specimen. The pores from Figure 12(a2,b3) had bar-type pores parallel to the long side of the specimens, which came from the gap between the infill and the perimeter as displayed in Figure 10(a2,b2) and Figure 11a. However, the results from Figure 12(c1,c3) showed rod-like pores in the vertical direction caused by the gaps between the layers (shown in Figure 10(c3,c4) and Figure 11b). For this reason, the porosity from printing also appeared in the final metal parts as well. The printing directions also played an essential role in pore formation. Therefore, it is vital to control the shape of pores during the printing process.

## 4. Conclusions

In this experiment, we produced metal specimens using bronze/PLA hybrid filament coupled with FFF 3D printing. We demonstrated the density change and dimension/weight shrinkage of sintered parts by reversing the sintering directions on cubes. In addition, we focused on the tensile stress and porosity of metal parts by researching printing/sintering orientations on tensile specimens. The methods and results of this work could be a reference point that contributes to the future improvement of sintered metal parts properties research. The conclusions of this study can be summarized as follows:

1. Sintering orientations had only minor effects on shrinkage, density, and porosity. The results from different sintering directions were similar. Shrinkage in the layer direction was lower than in the *x* and *y* directions. The density of the parts was increased by about 6.6 g/cm^3^ after sintering. The porosity was independent of the sintering direction. Parts produced in the “upright” sintering direction resulted in weakness leading to fracturing.
2. The printing orientations played an important role in tensile stress and porosity. “Upright” printed specimens were weak. Conversely, “on-edge” printed specimens had the best tensile stress at about 190 MPa. In addition, porosity occurred on the surfaces of the parts that were parallel to the printing bed. The pore volume was dependent on the area of the horizontally printed surfaces.
3. The best printing-sintering combination was “on-edge-flat”. The tensile stress and surface porosity supported these conclusions.

Towards the goal of producing higher-quality metal parts we will focus on minimizing the porosity of the printed parts in the future. The potential future research could focus on extending sintering time appropriately, increasing the percentage of metal powder in the hybrid filament, and using advanced materials to produce metal/polymer composite filament [35,36,37].

## Figures and Tables

**Table 1:** Composition of bronze/PLA hybrid filament.

Composition	Metal			Synthetic Material		
	Copper	Tin	Phosphorous	PLA	2-Propenenitrile, Polymer with 1,3-Butadiene and Ethenylbenzene	Binding Additive
Content (wt. %)	75.99	8.84	0.17	15	trace	trace

**Table 2:** Printing parameters for green parts.

Parameter	Unit	Value
Nozzle diameter	mm	0.6
Layer thickness (first layer)	mm	0.2
Layer thickness (left layers)	mm	0.3
Nozzle temperature (first layer)	°C	215
Nozzle temperature (left layers)	°C	210
Printing bed temperature	°C	60
Infill percentage	%	100
Flow degree	%	100
Printing speed	mm/s	70
Extrusion rate	mm^3^/s	4.9

**Table 3:** Combinations for printing and sintering directions of tensile simples.

Test Number	Printing Direction	Sintering Direction
1	Flat	Flat
2	Flat	On-edge
3	Flat	Upright
4	On-edge	Flat
5	On-edge	On-edge
6	On-edge	Upright
7	Upright	Flat
8	Upright	On-edge
9	Upright	Upright

**Table 4:** Shrinkage for dimension and weight and density of sintered metal cubes in five sintering directions.

Sintering Direction	Dimension Shrinkage (%)			Weight Shrinkage (%)	Density (g/cm^3^)	
	*x*-Axis	*y*-Axis	*z*-Axis		Before Sintering	After Sintering
On *xy* surface	20.82	19.58	14.43	14.7	3.72	6.93
On *xz* surface	20.01	21.61	11.74	15.42	3.7	6.61
On *yz* surface	21.21	20.08	11.53	15.19	3.72	6.56
On-edge	21.18	21.61	12.82	14.84	3.71	6.63
On point	20.37	20.52	13.6	14.78	3.7	6.53

## Data Availability

The data used to support the findings of this study are available from the corresponding authors upon request.
